# Fatty Acid Composition of Dry and Germinating Pollen of Gymnosperm and Angiosperm Plants

**DOI:** 10.3390/ijms24119717

**Published:** 2023-06-03

**Authors:** Maria Breygina, Alexander Voronkov, Tatiana Ivanova, Ksenia Babushkina

**Affiliations:** 1 Department of Plant Physiology, Biological Faculty, Lomonosov Moscow State University, Leninskiye gory 1-12, Moscow 119991, Russia; 2 Timiryazev Institute of Plant Physiology, Russian Academy of Sciences, Botanicheskaya St. 35, Moscow 127276, Russia; voronkov_as@mail.ru (A.V.);

**Keywords:** pollen tube growth, ROS, fatty acids, plant reproduction, lipids, pollen coat

## Abstract

A pollen grain is a unique haploid organism characterized by a special composition and structure. The pollen of angiosperms and gymnosperms germinate in fundamentally similar ways, but the latter also have important features, including slow growth rates and lower dependence on female tissues. These features are, to some extent, due to the properties of pollen lipids, which perform a number of functions during germination. Here, we compared the absolute content and the fatty acid (FA) composition of pollen lipids of two species of flowering plants and spruce using GC-MS. The FA composition of spruce pollen differed significantly, including the predominance of saturated and monoene FAs, and a high proportion of very-long-chain FAs (VLCFAs). Significant differences between FAs from integumentary lipids (pollen coat (PC)) and lipids of gametophyte cells were found for lily and tobacco, including a very low unsaturation index of the PC. The proportion of VLCFAs in the integument was several times higher than in gametophyte cells. We found that the absolute content of lipids in lily pollen is almost three times higher than in tobacco and spruce pollen. For the first time, changes in the FA composition were analyzed during pollen germination in gymnosperms and angiosperms. The stimulating effect of H_2_O_2_ on spruce germination also led to noticeable changes in the FA content and composition of growing pollen. For tobacco in control and test samples, the FA composition was stable.

## 1. Introduction

Pollen grains (PGs) are unique reproductive structures found in all seed plants, including both gymnosperms and angiosperms. They ensure the delivery of male gametes to the female gametophyte via a pollen tube (PT). The structure and main functions of PGs and PTs in the two plant groups are fundamentally similar, but there also are important differences, including, for example, growth rate, pollen wall construction, and the ability to interact with pollinators [1]. Undoubtedly, the systematic position of coniferous plants and the peculiarities of their reproduction are reflected in the biochemical composition of pollen grains. It should be noted that, in comparison with leaves, seeds, and other somatic tissues, the physiology of male gametophytes has been studied to a lesser extent: the majority of articles on conifers’ pollen germination appeared in the last two decades [1,2,3,4]. As for pollen biochemistry, there are several classic works on individual species, such as *Pseudotsuga menziesii*, *Pinus ponderosa*, *Picea pungens*, and *Cycas revoluta* [5,6,7,8].

PGs contain several lipidic structures, which play a key role in their development. The thick extracellular pollen wall, exine, is largely synthesized from acyl lipid and phenylpropanoid precursors, which together form the exceptionally stable biopolymer sporopollenin [9,10]. An additional extracellular lipidic matrix, pollen coat (PC), covers the interstices of the exine and performs functions in pollen dispersal and pollen–stigma recognition [11]. Two types of PC material exist in angiosperms: pollenkitt is the most common adhesive material present around PGs of almost all entomophilous angiosperms, whereas tryphine seems to be restricted to Brassicaceae [12]. In gymnosperms, no pollenkitt is produced (including *Gnetum gnemon*, which has a relatively advanced reproductive system with double fertilization [13,14]).

The coat lipids are not only important to protect pollen during the desiccation stage and transport, but are also involved in pollen attachment to stigma and the following interaction and signaling processes [12,15,16]. Another important function is less studied and includes attraction, feeding, and decontamination of pollinating insects. Fatty acids (FAs) are important in the reproduction, development, and nutrition of honeybees; the latter is provided mainly by palmitic (16:0) and oleic (9–18:1) acids [17]. Pollen with a high content of lauric (12:0), myristic (14:0), linoleic (9,12–18:2), and α-linolenic (9,12,15–18:3) acids, which are believed to inhibit the growth of spore-forming bacteria and other microbes that inhabit the brood combs of beehives. The bactericidal and antifungal properties of pollen are important for colony hygiene [17,18].

PC, as well as plant cuticle, has a high percentage of very-long-chain lipids (VLCL). In *Arabidopsis*, long chain lipids of tryphine were reported to be essential for proper pollen–stigma communication, resulting in pollen hydration on the dry stigma [19]. A substantial reduction in tryphine lipids in this species compromised pollen germination and, consequently, resulted in male sterility [20]. Jessen et al. investigated the role of two long-chain acyl-CoA synthetases (LACSs) in the production of tryphine lipids. Lacs1lacs4 knock-out mutants showed significant reductions in PC lipids and were conditionally sterile [20]. On the other hand, PC is not so important for pollen germination in *Crocus*, which also has a dry stigma: PGs with pollenkitt washed off after being deposited on the stigma emitted tubes that penetrated the papillae as effectively as that of untreated pollen [21].

In angiosperms, sporopollenin and PC precursors are both synthesized in tapetum—a specialized layer of nutritive cells within the anther—under the control of the sporophytic genome, but at different stages of development [10]. Sporopollenin precursors are exported from tapetum cells and deposited onto the surface of PGs [15]. Later during pollen development, the tapetal cells rupture, setting previously accumulated lipids and proteins free; these tapetum-derived substances overlay the exine and form the PC. During the last period of development, pollen also accumulates internal substances including polar lipids in the form of densely packed membranes, and neutral lipids such as triacylglycerols (TAGs) in lipid droplets (oil bodies) [15]. TAG synthesis is highly important for pollen development: thus, in *Arabidopsis*, a knockout mutant line, *dgat1-1/dgat1-1PDAT1/pdat1-2*, was almost unable to transmit mutant alleles for both genes through the male gametophyte. These plants produced close to 50% pollen that did not contain TAG, and its development was aborted after the microspore stage [22]. The synthesis of intracellular lipids in the PG is controlled by the gametophytic genome [11]. Therefore, the mature pollen contains two types of lipids: sporophyte-derived PC lipids and gametophyte-derived intracellular and membrane lipids [15]. During germination, the ratio of these groups changes: the proportion of membrane lipids, which are required in large quantities for PT growth, increases [15]. In angiosperms, sugars are taken up from the female apoplast and metabolized into lipids at a fast pace [23]. In tobacco pollen, the abundance of proteins involved in FA synthesis increases during PT growth until they make up ~1% of all proteins [24]. However, changes in the FA composition during germination are poorly studied: one recent study focused on multi–*omics* in pollen tubes (growing for more than 3 h) and described changes provoked by heat stress [25]; another study was dedicated to glycerolipid dynamics during pollen germination and pollen tube growth, and substantial changes in this group of lipids were found [26]. Both investigations were performed on the same object—tobacco. Important questions that we raised in this study were: (a) are dynamics of the FA composition similar in germinating pollen of coniferous and flowering plant; and (b) can factors that positively influence the efficiency of germination affect the FA composition of germinating pollen?

## 2. Results

### 2.1. Fatty Acid Composition of Spruce Pollen

**Figure 1:**
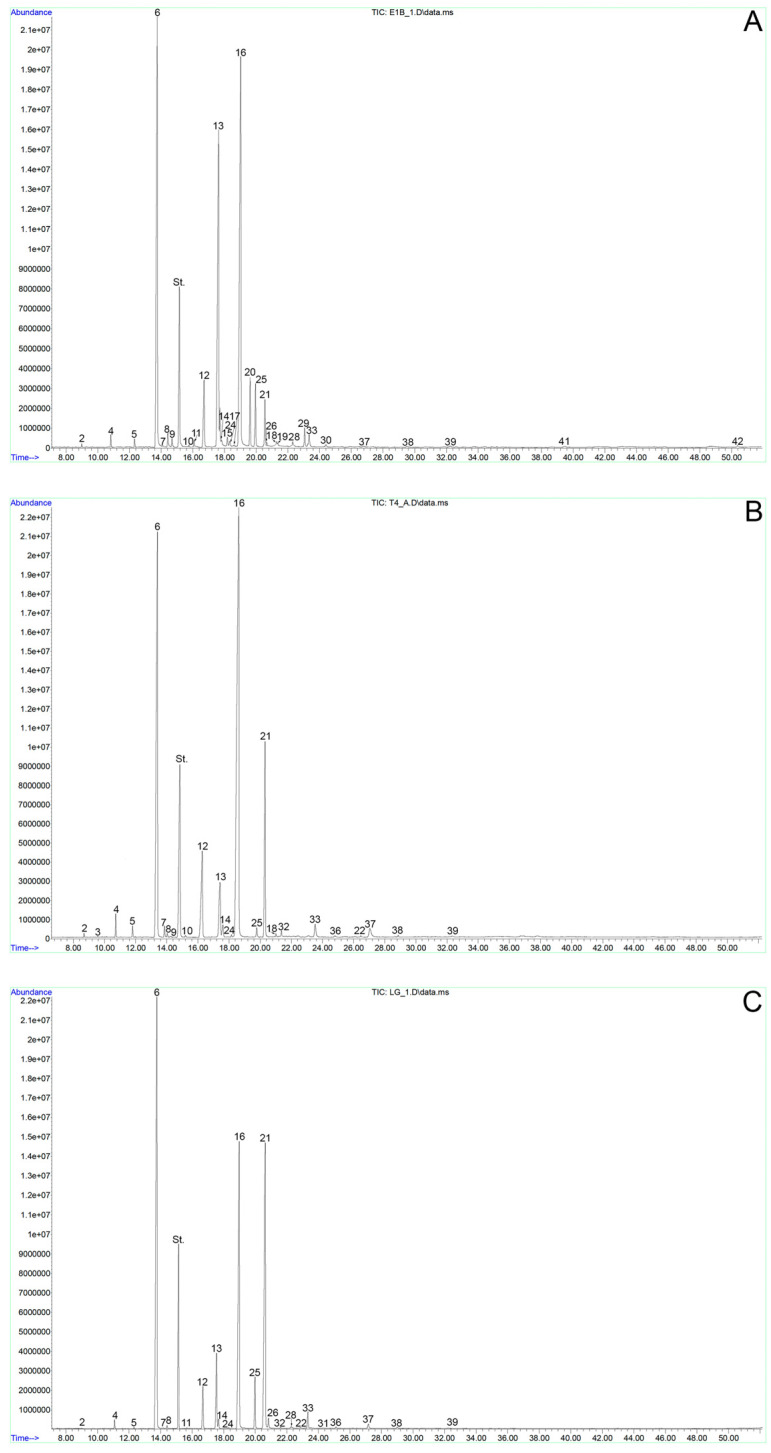
Chromatograms of FAMEs of spruce pollen (**A**) and PC-free pollen of tobacco (**B**) and lily (**C**). Numerals denote FAs according to their number in Table S1.

The total lipids of spruce pollen were represented by 31 types of individual C_12–30_ FAs. The major ones were 16:0, 9–18:1, and 9,12–18:2 acids (Table 1, Figure 1A).

**Figure 2:**
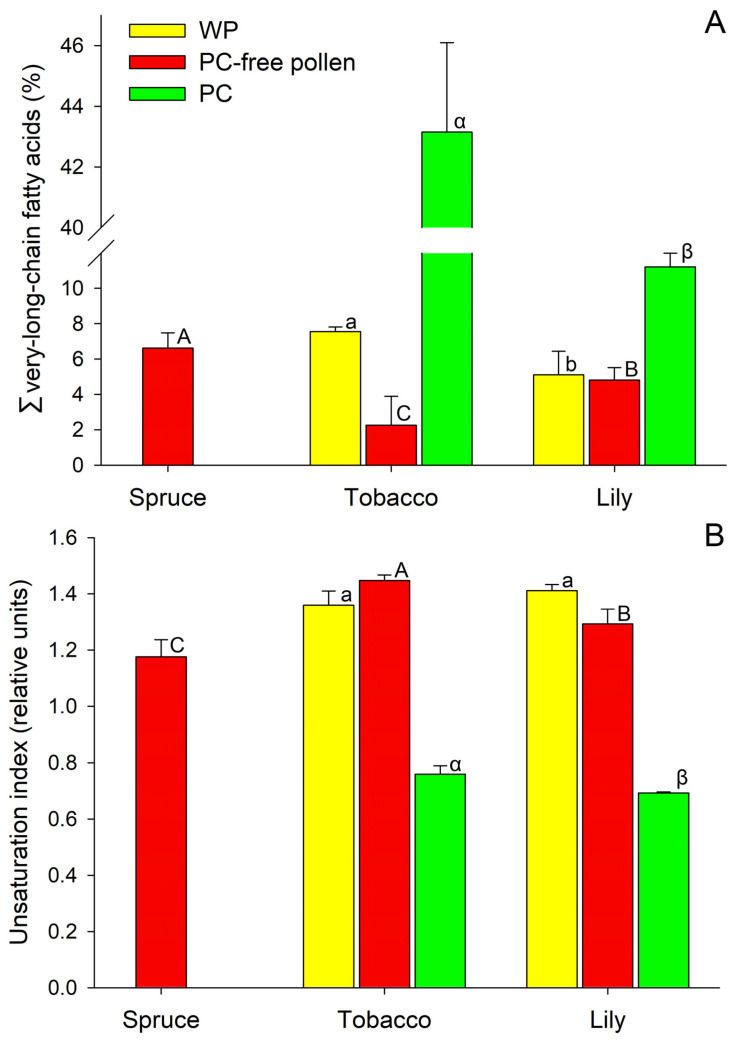
Sum of very-long-chain fatty acids (**A**) and unsaturated index (**B**) in pollen of spruce, tobacco, and lily. The data are presented as the means ± SEM. Statistical analysis was performed using one-way ANOVA followed by post hoc analysis using Tukey’s honest significant difference for unequal N tests. Different letters show significantly different values for WP (a, b), PC-free pollen (A, B, C), PC (α, β). Mean values were considered significantly different at *p* < 0.05. Analysis was performed separately for WP (whole pollen), PC (pollen coat), and PC-free pollen (washed with hexane).

Additionally of note is a significant proportion of stearic (18:0), pinolenic (5,9,12–18:3), and 9,12,15–18:3 FAs. The relative content of other FAs did not exceed 1% of the total amount. Very-long-chain FAs (VLCFAs—with more than 20 C atoms in the chain) of spruce pollen lipids made up 6.62% of the total FA content (Figure 2A), 2.71% of which accounted for arachidic FA (20:0) (Table 1).

**Figure 3:**
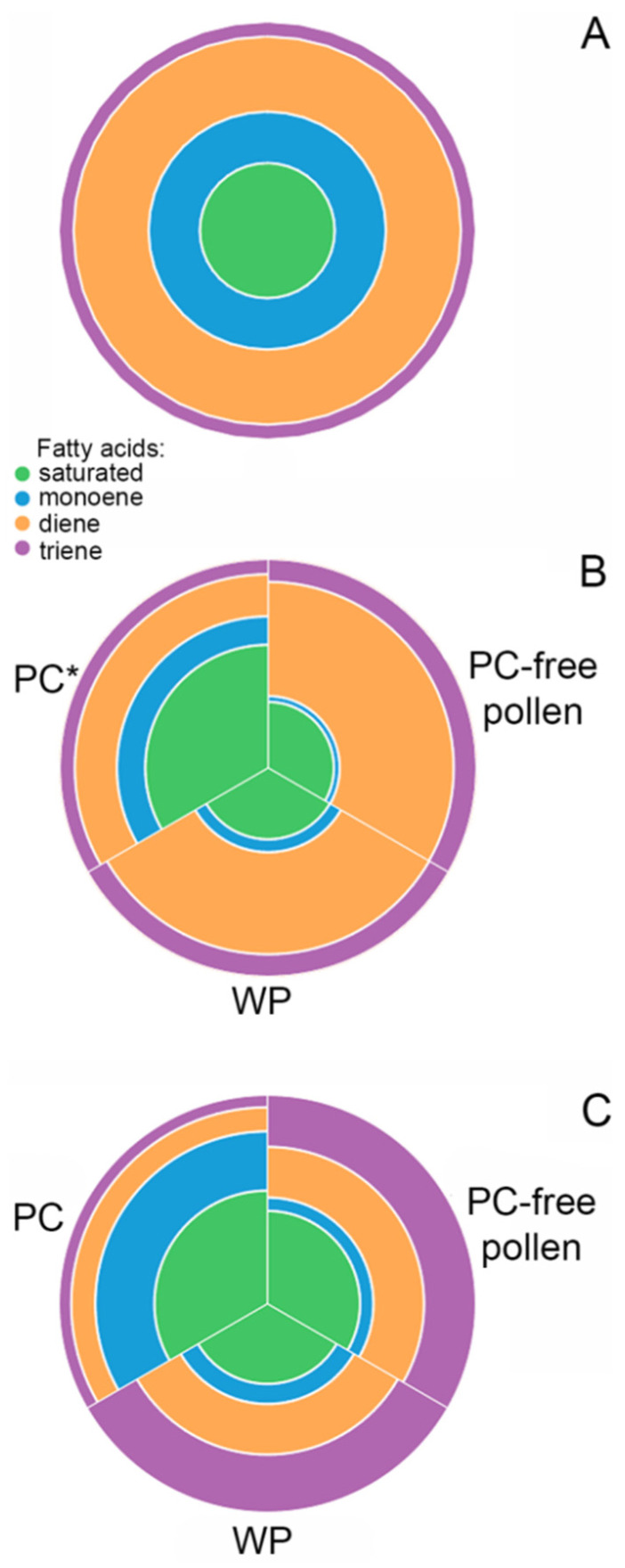
The ratio of saturated and groups of unsaturated (mono-, di-, triene) fatty acids in pollen of spruce (**A**), tobacco (**B**) and lily (**C**) in % of the total FAMEs. *—tetraene also found (0.02%).

The unsaturation index (UI) of spruce pollen lipids was 1.176 (Figure 2B); such a low value can be explained by a large proportion of saturated and monoene FAs and a low percentage of triene FAs (Figure 3A). 

### 2.2. Fatty Acid Composition of Tobacco Pollen

Since tobacco and lily pollen, unlike spruce pollen, is covered with a PC whose composition is markedly different from the internal parts of the male gametophyte. We considered three samples which were analyzed separately: whole pollen (WP), PC, and PC-free pollen (with PC washed off with hexane).

The total lipids of tobacco WP were represented by 32 types of individual C_10–28_ FAs. The major ones were 16:0, 9,12–18:2, and 9,12,15–18:3 acids (Table 1, Figure 1B). There was also a significant proportion of 18:0, 9–18:1, behenic (22:0), and lignoceric (24:0) FAs. The relative content of the rest did not exceed 1% for each FA. VLCFAs accounted for ≈7.6% of the total FA and were represented by 12 individual FAs, with 3.77% belonging to 22:0 (Figure 2A).

**Figure 4:**
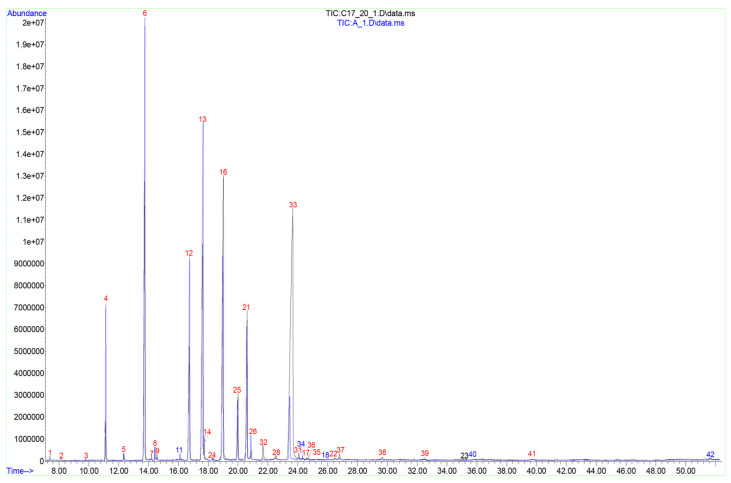
Chromatograms of pollen coat (PC) FAMEs of tobacco (black) and lily (blue). Numbers indicate FAs according to their number in Table S1: red—FAs are identified in PC of both tobacco and lily pollen, black—FAs are identified only in tobacco PC, blue—FAs are identified only in lily PC.

In PC-free tobacco pollen, the number of individual C_12–26_ FAs decreased to 24 (Figure 4). The main FAs were the same as in WP: palmitic, linoleic, and α-linolenic (Table 1); these three acids accounted for ≈92% of the total amount. The relative amount of palmitoleic acid decreased by three times. The relative content of VLCFAs was ≈2.25% of all FAs, which was several times less than in WP (Figure 2A).

The total lipids of PC contained 30 individual C_10–28_ FAs. The main acids were 16:0 (relative content two times less than in WP), 9–18:1 (almost three times more than in WP), 9,12–18:2 (≈three times less than in WP) and 22:0 (this VLCFA dominated and accounted for almost a third of all PC FAs) (Table 1). It is important to note the differences in the lipid components of PC relative to WP: the content of minor FAs: 18:0, 20:0, eicosadienoic (11,14–20:2), heneicosanoic (21:0), tricosanoic (23:0), and 24:0 increased several times. At the same time, the proportion of 9,12,15–18:3 decreased (Table 1). Only in PC was the presence of a rare α-parinaric (9,11,13,15–18:4; PubChem CID 5460995) acid belonging to the group of conjugated FAs registered (Table 1; Figures S1 and S2) [27]. Due to the significant differences described above, there was more than a five fold increase in the proportion of VLCFAs compared to WP samples: up to 43.15% (Figure 2A).

Shifts of UI reflect the differences in the proportion of FAs with different numbers of double bonds in lipids of tobacco WP, PC-free pollen, and PC components (Figure 2B). The lowest lipid UI was observed in PC—0.759, which is a consequence of the high proportion of saturated and monoene FAs and the low proportion of dienes and trienes (Figure 3B). The UI of total lipids of WP and PC-free pollen did not differ significantly and were 1.359 and 1.447, respectively (Figure 2B).

### 2.3. Fatty Acid Composition of Lily Pollen 

The total lipids of lily WP were represented by 34 types of individual C_10–30_ FAs. The major FAs were 16:0, 9,12–18:2 and 9,12,15–18:3; their relative content was more than 80% of the total (Table 1, Figure 1C). Note the minor content of 18:0, 9–18:1, and 20:0 acids; their impact did not exceed 1% (Table 1). VLCFAs accounted for 5.11% of total FAs and consisted of 15 individual species, with 2.4% belonging to 20:0 acid (Table 1; Figure 2A).

After PC components were washed off, the number of individual types of C_12–26_ FAs in lily pollen decreased to 24. The relative content of the major FAs did not change significantly, and they remained the same: palmitic, linoleic, and α-linolenic (Table 1). 18:0, 9–18:1 and 20:0 were present in minor amounts (Table 1). VLCFAs were represented by 10 individual species and accounted for 4.81% of the total identified FAs (Figure 2A).

The total lipids of lily PC contained 34 types of individual C_10–30_ FAs (Figure 4). The main FAs were 16:0, 18:0, 9–18:1, and 9,12–18:2 (Table 1). 12:0, 14:0, 20:0, gadoleic (11–20:1), 22:0, and 24:0 acids were registered in minor amounts in PC components (Table 1); the proportion of each remaining FA was less than 1% of the total. VLCFA accounted for 11.24% of the total PC FAs (Figure 2A), which is more than two times higher than in WP and PC-free pollen. Some minor FAs were registered only in PC components: capric (10:0), tridecanoic (13:0), 14-hexadecenoic (14–16:1), and others (Table 1).

UI of total lipids was calculated for WP, PC-free pollen, and PC components (Figure 2B). This indicator was close in WP (1.411) and after PC washout (1.293), which can be explained by a large proportion of polyene FAs. The UI of PC lipids was 0.692: saturated FAs dominated, and among the unsaturated FAs, monoene FAs accounted for the largest part (Figure 3C). 

### 2.4. The Lipid Absolute Contents of Spruce, Tobacco and Lily Pollen

Absolute lipid content in spruce pollen and tobacco PC-free pollen did not differ significantly (Table 1). At the same time, in PC-free lily pollen, it was almost three times higher than in the two other species (Table 1). Lily PC also contained significantly more lipids than tobacco PC (Table 1).

### 2.5. The Effect of Hydrogen Peroxide on Tobacco and Spruce Pollen Germination

To identify possible changes in the FA composition of pollen during germination, we decided to consider both germination on a standard incubation medium (control) and germination in the presence of a physiological regulator. The effects of toxic substances that reduce germination were outside the scope of this study. Therefore, we decided to test the effect of hydrogen peroxide, which was reported to affect pollen physiology positively [3,28,29].

**Figure 5:**
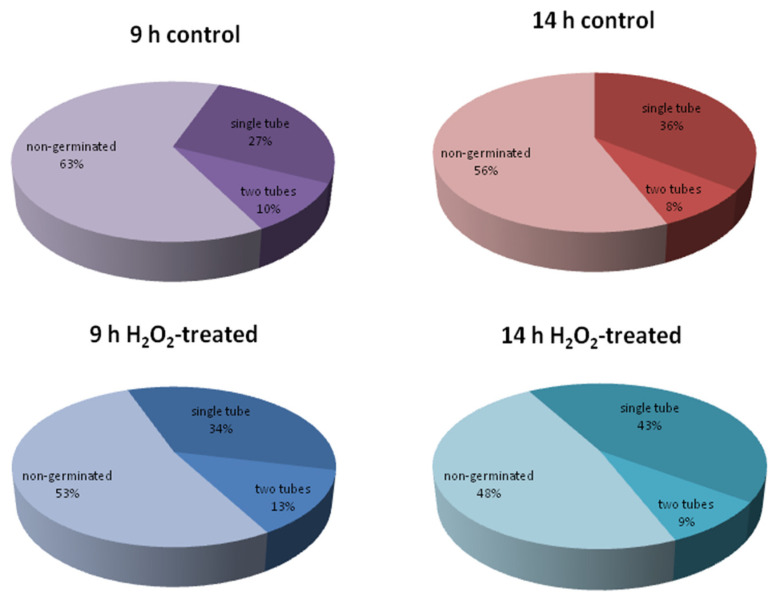
The effect of hydrogen peroxide on the germination of spruce pollen grains after 9 and 14 h of incubation. Means are presented. For each diagram, four suspensions were counted (*n* = 4), 400 to 900 cells in each. The level of germination in H_2_O_2_-treated samples significantly differs from the control at both times according to Mann–Whitney test, *p* < 0.05.

We found that 100 µM H_2_O_2_ stimulated pollen germination in spruce, which was detected at both time points (Figure 5): at 14 h, the percent of germinated grains was 44 ± 3 and 52 ± 3 for control and H_2_O_2_, respectively; at 9 h, this difference was more pronounced as the total germination was lower (38 ± 2 and 48 ± 3, respectively). Next, we looked at whether the detected change in germination efficiency was reflected in the FA composition. 

The same concentration of peroxide did not affect the efficiency of tobacco pollen germination: after one hour, it was 33.5 ± 2 and 36 ± 3 for control and H_2_O_2_, respectively (*n* = 4).

### 2.6. Dynamics of the Fatty Acid Composition and Lipid Absolute Content of Spruce Pollen during Germination with or without H_2_O_2_

We used two time points to monitor the germination process in spruce pollen. These points were chosen according to our previous studies [30,31], in which we found that after 9 h, exine forms one or two large gaps through which the pollen tube(s) comes out. At this time point, oxygen consumption and cytosolic pH reach their maximum. Nine hours is, therefore, considered the point of germination. At 14 h, the length of an average pollen tube reaches the diameter of pollen grain—this point is considered ongoing polar growth.

During germination and growth of spruce pollen in standard cultivation medium, no significant differences were recorded among the major FAs; only the content of 20:0 was significantly higher at the germination point (9 h). There were significant differences in the content of minor FAs: 12:0, palmitoleic (9–16:1), rumenic (9,11–18:2), 11–20:1, sciadonic (5,11,14–20:3), dihomo-γ-linolenic acid (8,11,14–20:3), pentacosylic (25:0) (Table S1). Some new FA or isomers appeared during pollen incubation. Thus, in growing spruce pollen tubes *cis*-6,11-eicosadienoic (6.11–20:2) was registered; during germination and growth, we did not detect melissic (30:0) FA, which was found in dry pollen.

When H_2_O_2_ was added to the cultivation medium during spruce pollen germination, the composition of lipid FAs changed. Among the major FAs, the relative amount of saturated 18:0 and 20:0 significantly decreased, but the content of unsaturated 9–18:1 and 9,12–18:2 increased. Peroxide addition significantly reduced the content of pentadecanoic (15:0), 7,10-hexadecadienoic (7,10–16:2), *cis*-10-heptadecenoic (10–17:1), 10,12-octadecadienoate (*trans*, *cis*-10,12–18:2), and 9,11–18:2, nonadecylic (19:0) FAs. Qualitative changes were also registered: 6,11–20:2 and 30:0 FAs were detected in peroxide-treated samples (Table S1). However, all H_2_O_2_-induced changes did not have a significant effect on UI, which was 1.123 and 1.214 for control and treated samples, respectively.

When H_2_O_2_ was added to the cultivation medium during PT growth, changes affected only minor FAs. The relative content of 10–17:1, 10,13-octadecadienoate (10,13–18:2), 11–20:1, and 8,11,14–20:3 FAs significantly decreased while 6,11–20:2 and 25:0 FAs significantly increased. Additionally, H_2_O_2_ initiated the appearance of 30:0, which was absent in control PT samples (Table S1). UI did not change significantly (1.188 and 1.236). 

During spruce pollen germination and growth, the absolute content of lipids did not change. At the same time, the addition of H_2_O_2_ to the cultivation medium led to a significant increase in the content of lipids (Table S1).

### 2.7. Dynamics of the Fatty Acid Composition and Lipid Absolute Content of Tobacco Pollen during Germination with or without H_2_O_2_

Tobacco germinates in vitro much faster than spruce, so the corresponding points were shifted and were 1 h for germination and 3 h for tube growth. No significant differences were found in the qualitative or quantitative content of the major and even minor FA between all time points and control/treated pollen (Table S1). Only superminor FAs were affected: 10:0 was registered only at the germination stage, 13:0 was absent during germination in H_2_O_2_-treated suspensions and during PT growth, 10,13–18:2 was absent in dry pollen and appeared during germination and growth in all samples, 11,14–20:2 was recorded during pollen germination with H_2_O_2_ and during growth in control medium, 25:0 was absent during pollen germination and PT growth with H_2_O_2_, and cerotic (26:0) was present only in dry pollen and during control germination. In total, such changes affected no more than 1% of FAs of total lipids. The absolute content of lipids in tobacco pollen did not change during germination and the addition of growth both to the medium, with and without peroxide (Table S1).

## 3. Discussion

In this work, we considered several aspects of the lipid FA composition of pollen. On the one hand, we identified key differences between pollen FA compositions of coniferous and flowering plants. Since PC layer is absent in the pollen of gymnosperms, we compared it with PC-free pollen of flowering plants. Such a comparison includes two aspects: (1) gymnosperms have their own peculiarities of FA composition, characteristic of different tissues and organs [32,33]; (2) pollen has its own differences in FA composition from vegetative tissues of the same species [15].

For dry pollen, the set of major FAs was quite typical for pollen of Pinaceae: 16:0, 9–18:1, and 9,12–18:2 acids. For comparison, the major components in *Pseudotsuga* were 16:0, 9–18:1, and 9,12–18:2, whereas in *Pinus* they were 16:0, 18:0, 9–18:1, and 9,12–18:2 acids [34,35,36]. In a comparative study analyzing lipids and FAs of five species of pine, ginkgo, and alder, similar patterns were found: the proportion of saturated 16:0 and monoene 9–18:1 in all *Pinus* species was about two times higher than in angiosperm pollen (*Alnus japonica*). 9,12,15–18:3, on the contrary, was minor in pollen of all pine species (1–11%), while in alder it accounted for more than 26% [37]. The total contents of saturated fatty acids in three *Pinus* species was reported to vary from 51.27% (*P. sylvestris*) to 56.36% (*P. sibirica*) [35].

As for the less abundant FAs, in *Picea* pollen we found 5,9,12–18:3—one of Δ5 FAs—which has previously been found in the pollen of other gymnosperms, including *Pinus yunnanensis* [38], other *Pinus* species [35] and *Cycas revoluta* [6]; moreover, in the first case, its content decreased during storage and sterilization in parallel with the loss of pollen quality [38]. Gymnosperm seed lipids are also characterized by the presence of peculiar FAs containing Δ5-ethylenic bond, frequently referred to as Δ5-olefinic acids, with 18 or 20 carbon atoms, which are seldom encountered in angiosperms [39]. These acids are: taxoleic (5,9–18:2; PubChem CID 5312476) (Figure S3A), pinolenic (5,9,12–18:3; PubChem CID 5312495) (Figure S3B), coniferonic (5,9,12,15–18:4; PubChem CID 13751481), 5,11-eicosadienoic (5,11–20:2; PubChem CID 13751478), sciadonic (5,11,14–20:3; PubChem CID 445084) (Figure S3C), and juniperonic (5,11,14,17–20:4; PubChem CID 5312543) [40]. Half of these FAs were found in spruce pollen, which was a clear difference from pollen of tobacco and lily. Angiosperms, with a few exceptions such as Ranunculaceae, have lost the ability to synthesize such FAs. Hence, Δ5 FAs could be considered as an archaic FA marker. Among conifers, these FAs also occur with different frequencies: a few species that presented a low overall content of Δ5 FAs, grow in warm-temperate regions, compared to other pine species. It is hypothesized that Δ5-olefinic acids might be related to cold-acclimation [39]. Δ5 FAs are also present in lipids from algae and mosses, and little is known about their functions. In *Chlamydomonas*, two lines harboring independent mutant alleles of desaturase (CrDES) failed to synthesize 5,9,12–18:3 and were more sensitive to stress of endoplasmic reticulum than their parental lines [41]. However, there is no experimental confirmation of the protective function of 5,9,12,15–18:4 in higher plants.

VLCFAs made up 6.62% of the total FA content in *Picea* pollen, 2.71% of which accounted for 20:0. This is consistent with the data on FAs from leaves: relatively high amounts of VLCFAs were detected in different genera of Pinaceae (*Pinus, Picea, Pseudotsuga, Abies, Cedrus, Keteleeria*) and in Cupressaceae, in contrast with what was observed in angiosperms leaves [32]. VLCFAs possess distinctive biophysical properties in increasing lipid hydrophobicity, interdigitation in leaflets of lipid bilayer, and the transition from a fluid to a gel phase [42]. These unique properties allow VLCFAs to facilitate the generation and stabilization of the highly curved membrane domains required in the formation of highly specialized cells, such as the polarized ‘shmoo’ cell in yeast. Nothing is known about the participation of VLCFAs in pollen tube formation, since in flowering plants they are present in much greater quantities in the PC than in membranes. It can be assumed that they play an important role in polar growth in all seed plants, but in conifers their role is greater, since their content is higher. However, further research is needed to test this hypothesis.

Another notable difference between pollen lipids in spruce and the other two species studied is UI. For spruce pollen, it was significantly lower compared to tobacco and lily. One possible explanation is the growth rate, which is many times slower in the pollen of coniferous plants than the average of flowering plants [1]. Another possible explanation is the more severe germination conditions in the cone compared to a flower [43,44], and the respectively higher requirements for the resistance of spruce pollen membranes to unfavorable factors.

Considering the FA composition of tobacco and lily pollen, we confirmed and refined the patterns of lipid FA distribution in pollen of flowering plants reported for other angiosperm species [45,46]. For both species, PC was characterized by a radically different set of FAs. We found that in tobacco, the proportion of 9,12,15–18:3, which was dominant in PC-free pollen (54%), was significantly lower in PC (17%) while 22:0 acid grew from less than 1% to 30% (Table 1). The content of saturated FAs—18:0, 20:0, 21:0, 23:0, and 24:0—increased several times, which led to a more than five fold increase (Figure 3) in the proportion of VLCFAs (Figure 2A) and very low UI (Figure 2B). In lily, VLCFA in PC were more than two times higher than in PC-free pollen. Two FAs—saturated 16:0 and monoenoic 9–18:1—accounted for about 50% of the total PC FAs in this species. These results are in good agreement with data on other species, including those with a rather remote systematic position. Thus, in *Brassica napus*, PC is highly enriched medium- and long-chain saturated FAs while intracellular PG lipids are highly enriched in C_18_ polyunsaturates and, particularly, 9,12,15–18:3 [11]. However, the predominance of monoene and saturated FAs in PC is not characteristic of all angiosperm species: for example, in *Crocus vernus* pollenkitt, most FAs are unsaturated (9,12,15–18:3–53%) [21]. In *Arabidopsis*, PC has a high percent of VLCL which was shown to be essential for proper pollen–stigma communication, resulting in the hydration of the pollen on the dry stigma [19,20]. The differences in the FA composition between lipids of the PC and PG cells can be convincingly explained by the origin of these lipids: PC synthesis is controlled by the sporophytic genome, while intracellular lipids are synthesized by the gametophyte [15].

For PC of both species, we noted the presence of some rare minor FAs, which were absent in the washed pollen, including parinaric acid (9,11,13,15–18:4) in tobacco. In cell-free model systems, it was shown that the inclusion of polyunsaturated conjugated FAs, in particular 9,11,13,15–18:4, into lipid bilayers can lead to an increase in their resistance to flocculation caused by divalent cations [47,48] and drastically increase membrane dissipativity when exposed to UV irradiation [49,50]. Additionally, this FA has a strong fluorescence in UV region [51,52]. The significance of the accumulation of 9,11,13,15–18:4 in the PC requires further study. However, it can be assumed that this is an adaptive feature of pollen integuments to protect against ultraviolet exposure and other environmental factors. As a surface component with UV fluorescence, 9,11,13,15–18:4 can also attract pollinators. It should be noted that 9,11,13,15–18:4, as well as other superminor conjugated FAs (10,12–18:2, 9,11–18:2, and 9,11,13–18:3) identified in pollen, may be formed artificially from 9,12–18:2 and 9,12,15–18:3 FAs under alkaline conditions [53,54,55]. Thus, we analyze this result with caution; however, the standard procedure we used did not lead to the formation of conjugates in any studied samples (Table 1), so we can assume that 9,11,13,15–18:4 found in tobacco pollen is most likely of natural origin.

Analyzing the absolute content of lipids in pollen, we found that in lily it exceeds by several times that in tobacco and spruce; however, the proportion of surface lipids in the composition of whole pollen in tobacco is 2.5 times higher than in lily (≈12.5% and ≈5%, respectively). This indicates a high proportion of internal storage lipids in lily pollen. In classical works elucidating the ultrastructure of *Lilium* pollen tubes, lipid bodies have been described [56,57], which appear to contain the stored material necessary for the very rapid growth of the tube. At the same time, in tobacco, a significant proportion of lipids are located on the surface of the pollen grain and, presumably, are involved in the recognition and hydration of pollen on the stigma, which in this species is covered with a lipid-rich exudate [58].

In this study we did not limit ourselves to dry pollen but looked at the dynamics of the FA composition during germination and the effect of H_2_O_2_ on it, and eventually discovered the fundamental differences between spruce and tobacco. The main difference was that the FA composition and the absolute lipid content of tobacco pollen both in control and H_2_O_2_-treated samples were very stable. For tobacco, the lipidomes of pollen tubes have been studied under heat stress, and it was found that both glycerophospholipids and galactoglycerolipids increased in saturated acyl chains under heat stress, while triacylglycerols changed less in respect to desaturation but increased in abundance [25]. It is interesting to note that, in this study, the negative environmental factor changed the fatty acid composition of pollen tube lipids, and we obtained data on the same object on the complete stability of such composition under H_2_O_2_ treatment. This emphasizes that peroxide is a normal physiological factor and can be opposed to stress factors to which the pollen lipids are forced to adapt. At the same time, the profile of FAs in spruce pollen changed both in control (minor FAs) and H_2_O_2_-treated samples (both major and minor FAs). Among the major FAs, the relative amount of saturated 18:0 and 20:0 significantly decreased, but the content of unsaturated 9–18:1 and 9,12–18:2 increased, which affected the UI, although these changes were not statistically significant. An increase in the proportion of unsaturated FAs is in good agreement with an increase in the germination rate, which is more pronounced in 9 h samples. As for the stability of the FA composition of tobacco pollen, it can be speculatively considered an evolutionary acquisition, although this statement needs further research.

## 4. Materials and Methods

***Plant material.*** Male cones from *Picea pungens* Engelm. were collected in Moscow State University Botanical Garden and kept at 25 °C until the scales opened to shed pollen, which was then stored at −20 °C. Defrosted pollen samples were used for cultivation.

Plants of *Nicotiana tabacum* L. var. Petit Havana SR1 were grown in a climatic chamber (25 °C, 16 h light) in vermiculite. The plants were watered with standard salt solution [59]. Anthers were removed just before flower opening and dried at 25 °C for three days. Mature pollen was preserved at −20 °C. Defrosted pollen was rehydrated in a humid chamber at 25 °C for 2 h before cultivation.

*Lilium longiflorum* Thunb. var. White Heaven anthers were collected from flowers bought in a local store and desiccated at 30 °C for 4 d; dry pollen was stored at −20 °C. 

***Pollen germination efficiency*** was assessed after 9 and 14 h of cultivation at 25 °C in standard medium containing, for *Picea*: 0.3 M sucrose, 1 mM CaCl_2_ and 1 mM H_3_BO_3_ in 15 mM MES-Tris buffer pH 5.5; for tobacco: 0.3 M sucrose, 1.6 mM H_3_BO_3_, 3 mM Ca(NO_3_)_2_, 0.8 mM MgSO_4_, and 1 mM KNO_3_ in 25 mM MES-Tris buffer, pH 5.8. Germinated PGs were fixed with 2% paraformaldehyde in 50 mM Na-phosphate buffer, pH 7.4 for minimum 30 min at 4 °C. Between 500 and 900 PGs were counted for each sample.

***Lipid extraction.*** PC was washed off of lily and tobacco pollen with hexane (1:100 m/V, 2 × 1 min) and analyzed separately. About 500 mg pollen (with or without PC) were fixed separately in 50 mL boiling isopropyl alcohol for 1 h to preserve the native lipid composition. The samples were stored at +4 °C until extraction. To extract lipids, isopropanol was separated through a Schott glass filter into a 200 mL volumetric flask, and plant material was homogenized in a porcelain mortar and extracted as previously described with slight modifications [60]. Lipids were extracted three times sequentially in chloroform/methanol/H_2_O (30:20:1.7, by vol.) and chloroform/methanol/HNO_3_ (20:10:0.1, by vol.). All the extracts were transferred to the volumetric flask, neutral pH was adjusted with NH_4_OH, and the total volume was adjusted to 200 mL with isopropanol containing 0.001% ionol (butylated hydroxytoluene, Sigma-Aldrich, Saint Louis, MO, USA, 34750) as an antioxidant. The extracts were stored at +4 °C.

***Preparation and analysis of fatty acid methyl esters.*** FA methyl esters (FAMEs) were prepared according to the previously described method with slight modifications [61]. A total of 50 μg of margaric acid (17:0) (Sigma Aldrich, Saint Louis, MO, USA, H3500) was added to 50 mL of extract as an internal standard. The aliquot (50 mL) of the extract (or PC in hexane) was evaporated to dryness using a rotary evaporator under standard conditions, and saponification was carried out in a boiling solution of 4% NaOH in methyl alcohol/water (1:1, by vol.). Then, the sample was evaporated to dryness using a rotary vacuum evaporator. H_2_O (1–2 mL) was added to the dried sample and unsaponifiable FAs were washed out several times with hexane until clearness. Then, a few drops of methyl orange were added to the remaining water-soluble fraction, and it was acidified with 20% H_2_SO_4_ to a pink color. Then, FAs were extracted six times with hexane. The collected hexane was evaporated, 3 mL of methanol and a few drops of acetyl chloride were added to the sample, and it was boiled for 1 h. Then, the sample was again evaporated, 1–2 mL of H_2_O and a few drops of methyl orange were added, and methyl esters of fatty acids were extracted six times with hexane. Following this, the hexane was evaporated and 500 μL of benzene was added. The extract in benzene was pipetted onto a chromatographic plate, and a mixture of hexane/diethyl ether/glacial acetic acid (8:2:0.1, by vol.) was used as the mobile phase. When the front passed to the top of the plate, the plate was removed and air dried for 1–2 min. Then, the plate was treated with a 0.001% solution of 2′,7′-dichlorofluorescein in ethanol and air-dried for 5–7 min. The FAME-containing zones were visualized in UV light (λ = 365 nm). Then, the sorbent from the FAME-containing zone of chromatographic plate was removed using a scalpel and transferred to a Schott glass filter, and the FAMEs were eluted from the sorbent by washing out with hexane six times [62].

The FAMEs were analyzed via gas chromatography-mass spectrometry (GC-MS) on an Agilent 7890A GC (Agilent, Santa Clara, CA, USA) with a quadrupole mass detector Agilent 5975C fitted with a 60-m capillary column DB-23 (inner diameter 0.25 mm, thickness of stationary phase-(50%-cyanopropyl)–methylpolysyloxane—250 µm). The prepared FAMEs were separated under the following conditions: carrier gas, helium at 1 mL/min; sample volume, 1 µL; split ratio, 4:1 (in numerous analyses, splitless injection was used); evaporator temperature, 260 °C. The oven temperature program was as follows: from 130 to 170 °C at 6.5 °C/min, to 215 °C at 2.75 °C/min (25 min hold at this temperature), to 240 °C at 40 °C/min (30 min hold at 240 °C). The operational temperature of the mass detector was set to 240 °C, and the ionization energy was set to 70 eV. To identify individual FAME species, NIST and Wiley search libraries and MSD ChemStation software, G1701EAE.0200.493 (Agilent, Santa Clara, CA, USA) were used, and the relative retention time and equal chain length (ECL) value were calculated for each peak [63].

***UV/Vis-absorption curves of methyl esters of fatty acids*.**Two milligrams of FAMEs was dissolved in 1 mL of hexane, and the absorption spectra of the prepared solutions were measured at 200–500 nm using UV/Vis spectrophotometers (Shimadzu UV-2700, Kyoto, Japan or Hitachi 557, Tokyo, Japan).

***Data and Statistical Analyses.*** To characterize the unsaturation level of lipid FAs, the UI [64] were calculated. All experiments were performed in triplicate with at least 3–5 independent executions. The data are presented in tables and a graph as the means ± SEM. Statistical analysis was performed using one-way ANOVA followed by post hoc analysis using Tukey’s honest significant difference (HSD) for unequal N tests. Different letters and symbols show significantly different values. Mean values were considered significantly different at *p* < 0.05. For pollen germination experiments, significant difference was evaluated by OriginLab 9.7 software (Northampton, MS, USA) according to Mann–Whitney test, *p* < 0.05.

## 5. Conclusions

In this work, dynamics and specific features of *Picea* pollen lipids were revealed, both in a state of physiological dormancy and at two time points during germination. In standard cultivation medium, no significant differences were recorded among the major FAs, though minor ones changed; under the effect of H_2_O_2_, which stimulated germination, we found significant changes in the FA composition and absolute lipid content. *Picea* pollen is characterized by a low degree of lipid unsaturation and a high proportion of VLCFAs. Some rare FAs were identified in pollen for the first time, and their possible functions are discussed. We analyzed the FA composition of pollen of representatives of monocots and dicots with wet stigmas in detail (surface lipids and cells separately), which made it possible to compare new data with the results previously reported for plants with dry stigmas—*Crocus, Brassica*, and *Arabidopsis*—and clarify general patterns and discuss the differences.

## Figures and Tables

**Table 1:** Fatty acid composition and absolute content of lipids (esterified FAs, μmol/g) of spruce, tobacco and lily pollen (mass % of the amount of FAMEs). Since spruce pollen does not have a pollen coat, we compared it with washed pollen of tobacco and lily (column “PC-free pollen”.

No.	FA	Spruce Pollen	Tobacco			Lily		
			WP	PC-Free Pollen	PC	WP	PC-Free Pollen	PC
1	10:0	-	0.01 ± 0.01	-	0.08 ± 0.02β	trace ^#^	-	0.19 ± 0.01α
2	12:0	0.10 ± 0.01A	0.03 ± 0.01b	0.06 ± 0.02B	0.29 ± 0.04β	0.22 ± 0.08a	0.04 ± 0.01B	2.97 ± 0.04α
3	13:0	-	0.01 ± 0.01	0.01 ± 0.01	0.02 ± 0.01α	trace	-	0.03 ± 0.01α
4	14:0	0.53 ± 0.06A	0.49 ± 0.05b	0.42 ± 0.07A	1.22 ± 0.14β	0.75 ± 0.08a	0.44 ± 0.08A	5.56 ± 0.27α
5	15:0	0.30 ± 0.03A	0.36 ± 0.05a	0.29 ± 0.06A	0.26 ± 0.02α	0.10 ± 0.01b	0.08 ± 0.01B	0.28 ± 0.01α
6	16:0	23.12 ± 1.57C	24.91 ± 0.10b	26.93 ± 0.76B	11.65 ± 1.12β	30.25 ± 1.09a	37.35 ± 3.00A	23.68 ± 1.01α
7	7–16:1	0.57 ± 0.05A	0.53 ± 0.14a	0.42 ± 0.06B	0.50 ± 0.06α	0.25 ± 0.01b	0.17 ± 0.05C	0.55 ± 0.01α
8	9–16:1	0.23 ± 0.01A	0.14 ± 0.01b	0.12 ± 0.01C	0.13 ± 0.03β	0.23 ± 0.01a	0.19 ± 0.01B	0.25 ± 0.02α
9	14–16:1	0.44 ± 0.04A	0.07 ± 0.02a	0.02 ± 0.01B	0.12 ± 0.01α	0.01 ± 0.01b	-	0.04 ± 0.01β
10	7,10–16:2	0.01 ± 0.01A	trace	0.03 ± 0.01A	-	-	-	-
11	10–17:1	0.09 ± 0.02A	-	-	-	0.02 ± 0.01	0.01 ± 0.01B	0.07 ± 0.03
12	18:0	3.48 ± 0.26A	1.75 ± 0.23b	1.68 ± 0.17C	4.82 ± 0.07β	2.89 ± 0.37a	2.61 ± 0.14B	11.98 ± 0.90α
13	9–18:1	21.47 ± 0.94A	4.32 ± 0.83b	1.36 ± 0.09C	11.82 ± 0.91β	7.68 ± 0.53a	4.75 ± 0.26B	25.11 ± 1.18α
14	11–18:1	1.21 ± 0.28A	0.83 ± 0.03a	0.86 ± 0.03B	0.61 ± 0.09β	0.68 ± 0.19a	0.65 ± 0.05C	0.91 ± 0.11α
15	5,9–18:2	0.33 ± 0.10	-	-	-	-	-	-
16	9,12–18:2	33.79 ± 3.74B	47.47 ± 1.80a	54.10 ± 0.50A	17.46 ± 1.54α	23.97 ± 0.68b	24.04 ± 1.44C	10.99 ± 0.07β
17	10,13–18:2	0.05 ± 0.01	0.07 ± 0.02a	-	0.79 ± 0.10	0.04 ± 0.04a	-	trace
18	10,12–18:2	0.58 ± 0.09A	0.60 ± 0.75	0.36 ± 0.06A	-	trace	-	0.13 ± 0.01
19	9,11–18:2	0.73 ± 0.09	-	-	-	-	-	-
20	5,9,12–18:3	3.56 ± 0.49	-	-	-	-	-	
21	9,12,15–18:3	2.53 ± 0.28C	10.68 ± 1.64b	10.97 ± 0.51B	6.92 ± 0.39α	27.69 ± 0.80a	24.80 ± 0.84A	5.79 ± 0.42α
22	9,11,13–18:3	-	trace	0.02 ± 0.02A	0.08 ± 0.01α	trace	0.02 ± 0.01A	0.13 ± 0.05α
23	9,11,13,15–18:4	-	trace	-	0.02 ± 0.01	-	-	-
24	19:0	0.26 ± 0.16A	0.13 ± 0.01a	0.10 ± 0.03A	0.06 ± 0.01β	0.07 ± 0.01b	0.04 ± 0.02B	0.13 ± 0.02α
25	20:0	2.71 ± 0.15A	0.92 ± 0.21b	0.30 ± 0.05B	2.70 ± 0.14α	2.40 ± 0.41a	2.57 ± 0.34A	2.83 ± 0.17α
26	11–20:1	0.48 ± 0.05B	0.25 ± 0.15b	-	0.38 ± 0.01β	0.71 ± 0.02a	0.62 ± 0.01A	1.19 ± 0.05α
28	11,14–20:2	0.36 ± 0.06A	0.37 ± 0.03a	-	1.11 ± 0.14α	0.15 ± 0.02b	0.15 ± 0.03B	0.25 ± 0.08β
29	5,11,14–20:3	1.05 ± 0.14	-	-	-	-	-	-
30	8,11,14–20:3	0.01 ± 0.01	-	-	-	-	-	-
31	11,14,17–20:3	-	0.12 ± 0.09a	-	0.35 ± 0.02α	0.03 ± 0.01a	0.02 ± 0.01	0.08 ± 0.02β
32	21:0	-	trace	0.05 ± 0.03A	0.77 ± 0.09α	0.10 ± 0.05	0.09 ± 0.03A	0.18 ± 0.03β
33	22:0	0.86 ± 0.14A	3.77 ± 0.73a	0.87 ± 0.09A	29.77 ± 1.81α	0.87 ± 0.31b	0.89 ± 0.19A	3.71 ± 0.16β
34	13–22:1	-	-	-	-	trace	-	0.14 ± 0.01
35	13,16–22:2	-	0.20 ± 0.05	-	0.75 ± 0.08α	trace	-	0.11 ± 0.01β
36	23:0	-	0.16 ± 0.01a	0.08 ± 0.03A	1.40 ± 0.16α	0.14 ± 0.01a	0.06 ± 0.01A	0.47 ± 0.08β
37	24:0	0.76 ± 0.12A	1.59 ± 0.40a	0.93 ± 0.08A	5.29 ± 0.55α	0.42 ± 0.11b	0.35 ± 0.07B	1.34 ± 0.07β
38	25:0	0.07 ± 0.03A	0.09 ± 0.06a	0.01 ± 0.01B	0.13 ± 0.01α	0.05 ± 0.05a	0.02 ± 0.01B	0.09 ± 0.01β
39	26:0	0.16 ± 0.03A	0.13 ± 0.07a	0.01 ± 0.01C	0.47 ± 0.08α	0.08 ± 0.06a	0.04 ± 0.01B	0.54 ± 0.04α
40	27:0	-	-	-	-	trace	-	0.01 ± 0.01
41	28:0	0.15 ± 0.12	0.01 ± 0.01a	-	0.03 ± 0.01β	0.14 ± 0.24a	-	0.23 ± 0.05α
42	30:0	0.01 ± 0.01	-	-	-	0.02 ± 0.01	-	0.04 ± 0.01
FAs absolute content, µmol/g		151.11 ± 5.52B	160.85 ± 13.72b	136.59 ± 11.60B	19.88 ± 1.75β	580.67 ± 79.11a	430.68 ± 76.10A	28.24 ± 3.65α

^#^—<0.01%. The data are presented in tables and a graph as the means ± SEM. Statistical analysis was performed using one-way ANOVA followed by post hoc analysis using Tukey’s honest significant difference (HSD) for unequal N tests. Different letters show significantly different values. Mean values were considered significantly different at *p* < 0.05. Analysis was performed separately for WP (a, b), PC-free pollen (A, B), PC (α, β).

## Data Availability

The data presented in this study are available on request from the corresponding author. The data are not publicly available due to the University restrictions.

## Supplementary Materials

The following supporting information can be downloaded at: https://www.mdpi.com/article/10.3390/ijms24119717/s1.
