# Supplementary material for: Fatty Acid Composition of Dry and Germinating Pollen of Gymnosperm and Angiosperm Plants
Source: Int J Mol Sci. 2023 Jun 3;24(11):9717. doi: 10.3390/ijms24119717 (PMC10253635; doi:10.3390/ijms24119717)
Supplement: Supplementary file 1 [file ijms-24-09717-s001.zip › Supplementary Figures_MA.docx]

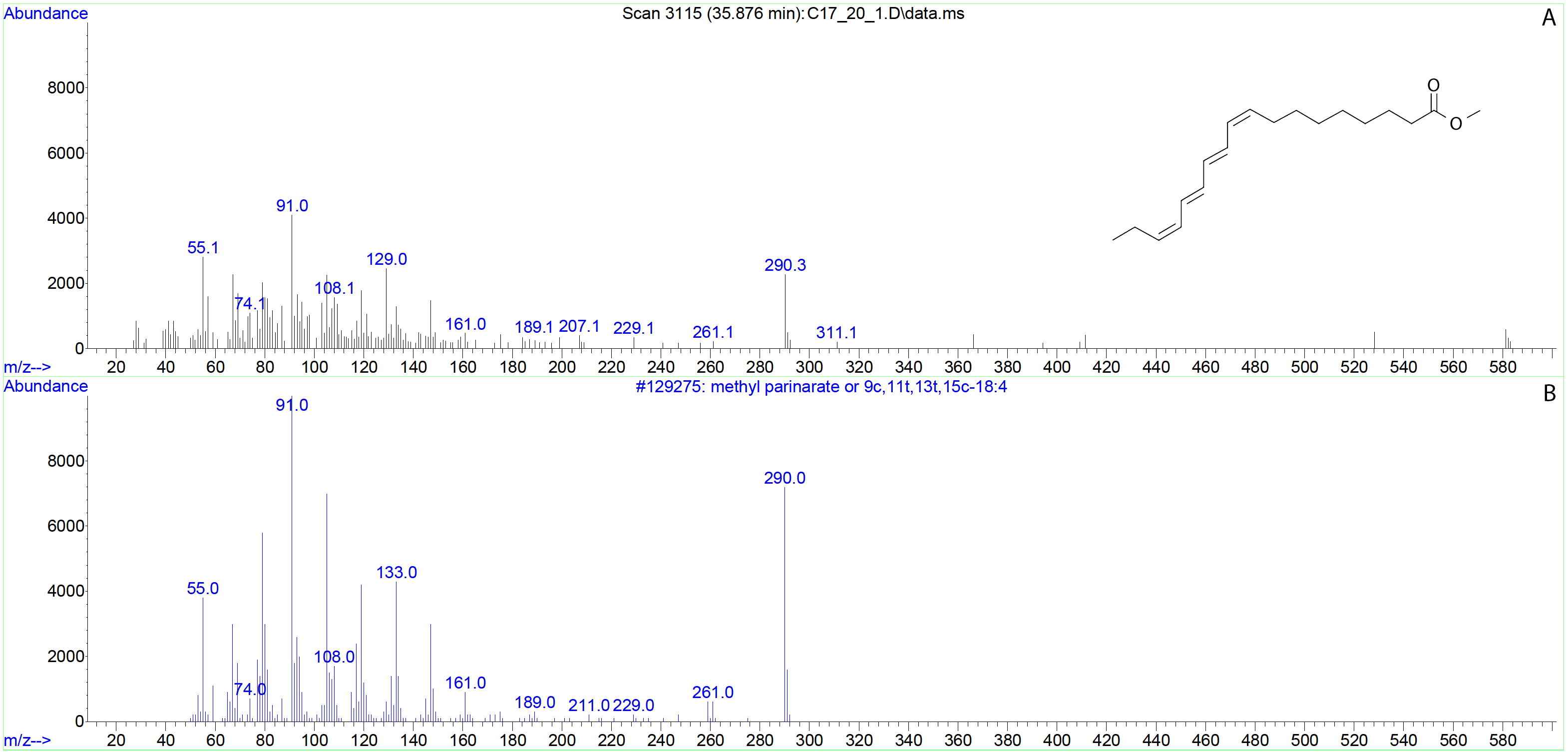


**Figure S1.** Mass spectra of α-parinaric (9,11,13,15-18:4) FAME in the sample of tobacco PC (**A**) and in NIST search libraries (**B**).


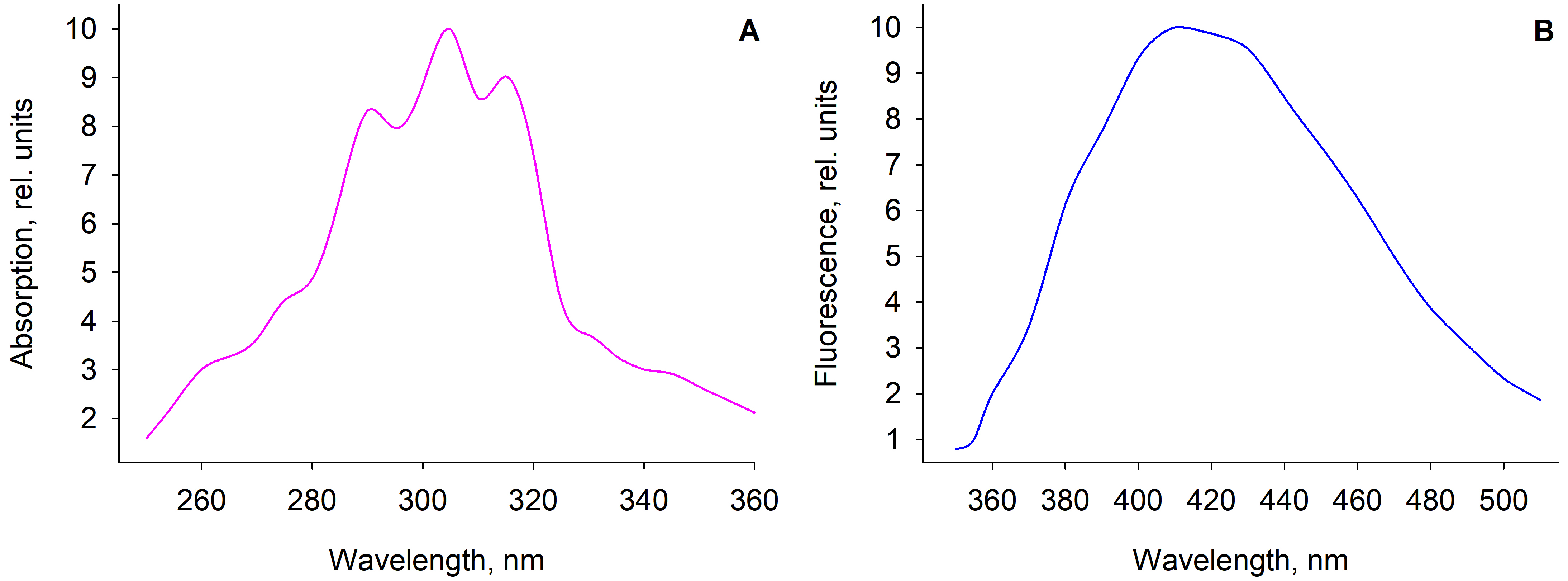


**Figure S2.** Absorption (**A**) and fluorescence (**B**) peaks typical for α-parinaric acid (9,11,13,15-18:4) of the extract of fatty acid methyl esters in hexane from tobacco pollen coat.


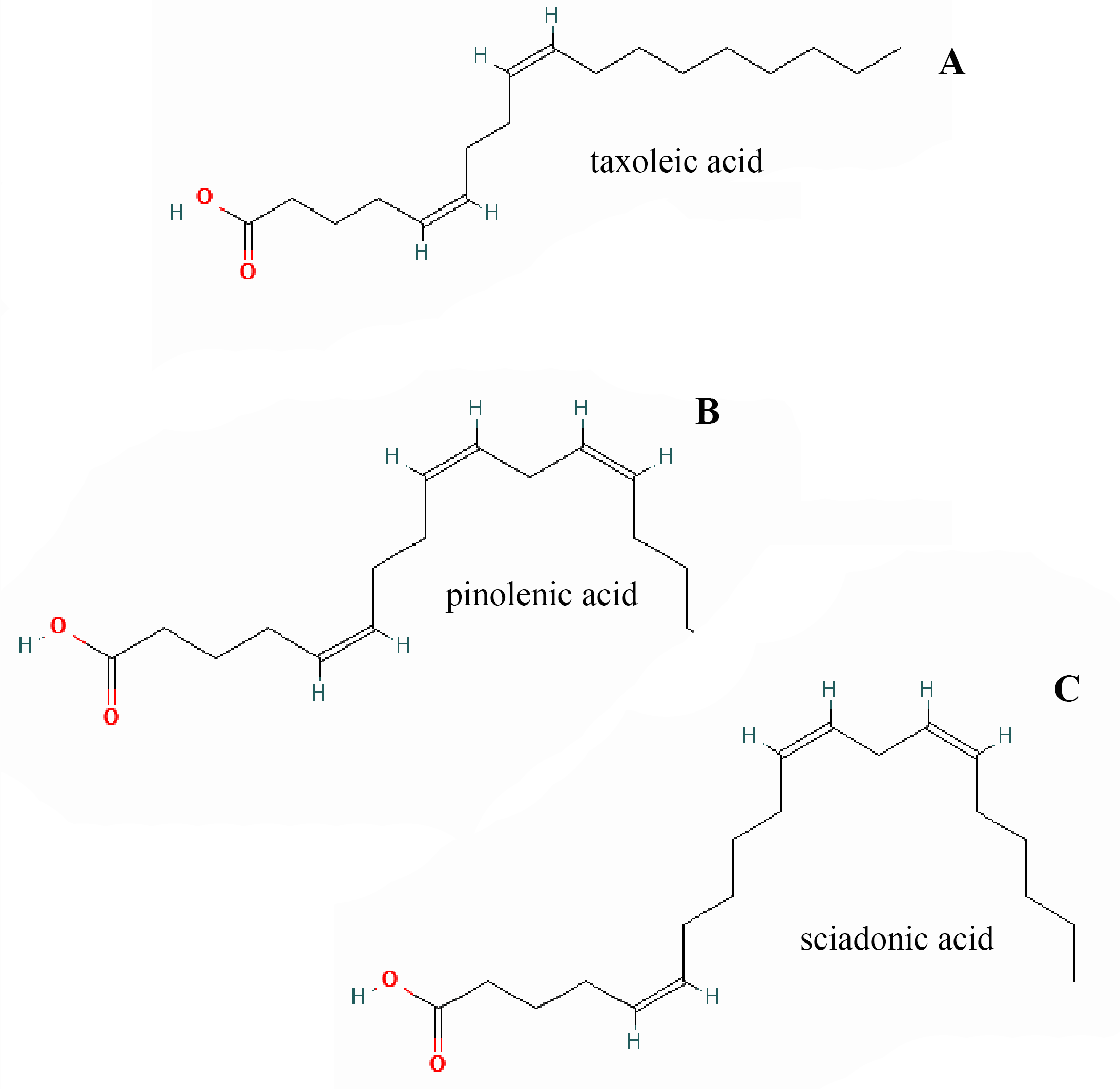


**Figure S3.** Structural formulas of some fatty Δ5 FAs found in spruce pollen lipids: 5,9–18:2 (**A**), 5,9,12–18:3 (**B**), 5,11,14–20:3 (**C**).
