# Supplementary material for: Fatty Acid Composition of Dry and Germinating Pollen of Gymnosperm and Angiosperm Plants
Source: Int J Mol Sci. 2023 Jun 3;24(11):9717. doi: 10.3390/ijms24119717 (PMC10253635; doi:10.3390/ijms24119717)
Supplement: Supplementary file 1 [file ijms-24-09717-s001.zip › Supplementary Table S1.pdf]

Table S1. The fatty acid composition and absolute content of lipids (esterified FAs, μmol/g) of spruce and tobacco pollen during germination with or without H_2O_2 (mass % of the amount of FAMEs).

№	FAs	Pollen	Control: Dry pollen	Control: Initial stage of germination	Control: PT growth	$+H_2O_2$: Initial stage of germination	$+H_2O_2$: PT growth
1	10:0	spruce	-	-	-	-	-
		tobacco	-	0.03±0.03a	-	0.01±0.01	-
2	12:0	spruce	0.10±0.01a	0.06±0.04a	0.05±0.02b	0.01±0.01A	0.04±0.02A
		tobacco	0.06±0.02a	0.04±0.01a	0.01±0.01b	0.06±0.04A	0.03±0.01A
3	13:0	spruce	-	-	-	-	-
		tobacco	0.01±0.01a	0.01±0.01a	-	-	-
4	14:0	spruce	0.53±0.06a	0.67±0.26a	0.60±0.19a	0.34±0.06A	0.40±0.06A
		tobacco	0.42±0.07b	0.41±0.04b	0.32±0.04a	0.42±0.18A	0.36±0.03A
5	15:0	spruce	0.30±0.03a	0.50±0.23a	0.40±0.15a	0.21±0.02A*	0.22±0.05A
		tobacco	0.29±0.06a	0.29±0.03a	0.24±0.02a	0.29±0.07A	0.27±0.03A
6	16:0	spruce	23.12±1.57a	23.13±1.18a	23.21±1.21a	21.35±0.56A	20.31±2.80A
		tobacco	26.93±0.76a	27.62±1.25a	25.90±1.59a	27.24±0.69A	26.75±0.37A
7	7-16:1	spruce	0.57±0.05a	0.93±0.51a	0.80±0.39a	0.37±0.07A	0.39±0.09A
		tobacco	0.42±0.06a	0.87±0.48a	0.30±0.01b	0.39±0.07A	0.36±0.05A
8	9-16:1	spruce	0.23±0.01ab	0.20±0.02b	0.25±0.01a	0.19±0.01A	0.21±0.02A
		tobacco	0.12±0.01a	0.12±0.01a	0.12±0.01a	0.13±0.03A	0.14±0.03A
9	14-16:1	spruce	0.44±0.04a	0.42±0.06a	0.40±0.02a	0.36±0.02A	0.37±0.01A
		tobacco	0.02±0.01a	0.02±0.01a	0.02±0.01a	0.02±0.01A	0.03±0.01A
10	7,10-16:2	spruce	0.01±0.01b	0.10±0.01a	0.03±0.01b	0.04±0.02A*	0.01±0.01A
		tobacco	0.03±0.01a	0.03±0.01a	0.02±0.01a	0.02±0.01B	0.04±0.01A
11	10-17:1	spruce	0.09±0.02a	0.18±0.11a	0.13±0.04a	0.02±0.01B*	0.06±0.02A•
		tobacco	-	-	-	-	-
12	18:0	spruce	3.48±0.26a	4.33±0.45	3.41±0.17a	3.36±0.07A*	3.34±0.84A
		tobacco	1.68±0.17a	1.72±0.20a	1.96±0.16a	3.24±2.22A	1.71±0.11A
13	9-18:1	spruce	21.47±0.94a	19.64±1.03	21.23±0.76a	22.47±0.56A*	21.77±0.30A
		tobacco	1.36±0.09a	1.25±0.16a	1.45±0.06a	2.21±1.29A	1.37±0.09A
14	11-18:1	spruce	1.21±0.28a	1.10±0.20a	1.29±0.07a	0.89±0.06A	1.01±0.33A
		tobacco	0.86±0.03a	0.83±0.07a	0.87±0.03a	1.33±0.95A	0.86±0.06A
15	5,9-18:2	spruce	0.33±0.10a	0.53±0.16a	0.43±0.04a	0.46±0.01A	0.52±0.09A
		tobacco	-	-	-	-	-
16	9,12-18:2	spruce	33.79±3.74a	31.32±2.28a	34.15±1.30a	35.60±0.53A*	35.31±1.60A
		tobacco	54.10±0.50a	53.29±0.28a	54.66±1.53a	50.97±5.00A	53.92±1.09A
17	10,13-18:2	spruce	0.05±0.01a	0.11±0.01b	0.16±0.02a	0.12±0.02A	0.10±0.01A•
		tobacco	-	0.07±0.04b	0.15±0.01a	0.10±0.03B	0.24±0.08A
18	10,12-18:2	spruce	0.58±0.39ab	0.50±0.21a	0.19±0.07b	0.20±0.05A*	0.21±0.04A
		tobacco	0.36±0.06a	0.36±0.05a	0.39±0.10a	1.10±1.01A	0.46±0.06A
19	9,11-18:2	spruce	0.73±0.41a	1.09±0.54a	0.02±0.01b	0.17±0.03A*	0.02±0.01B
		tobacco	-	-	-	-	-
20	5,9,12-18:3	spruce	3.56±0.49a	3.56±0.61a	3.73±0.15a	3.93±0.21A	4.26±0.77A
		tobacco	-	-	-	-	-
21	9,12,15-18:3	spruce	2.53±0.28a	2.55±0.38a	2.76±0.11a	2.74±0.16A	3.06±0.33A
		tobacco	10.97±0.51a	11.01±0.40a	11.04±0.35a	10.02±0.85A	10.92±0.26A
22	9,11,13-18:3	spruce	-	-	-	-	-
		tobacco	0.02±0.02a	0.05±0.01a	0.04±0.02a	0.09±0.02A	0.07±0.02A
24	19:0	spruce	0.26±0.16a	0.23±0.07a	0.25±0.11a	0.13±0.01B*	0.21±0.04A
		tobacco	0.10±0.03a	0.11±0.02a	0.11±0.01a	0.11±0.01A	0.13±0.02A
25	20:0	spruce	2.71±0.15a	3.67±0.09a	2.63±0.45b	2.85±0.11A*	2.80±0.54A
		tobacco	0.30±0.05a	0.30±0.06a	0.32±0.03a	0.33±0.08A	0.29±0.04A
26	11-20:1	spruce	0.48±0.05b	0.63±0.14ab	0.65±0.02a	0.79±0.06A	0.49±0.12B•
		tobacco	-	-	-	-	-
27	6,11-20:2	spruce	-	-	0.04±0.01	0.06±0.01A	0.11±0.03A•
		tobacco	-	-	-	-	-
28	11,14-20:2	spruce	0.36±0.06a	0.46±0.13a	0.39±0.09a	0.45±0.05A	0.42±0.04A
		tobacco	-	-	0.05±0.01	0.06±0.02A	-
29	5,11,14-20:3	spruce	1.05±0.14a	0.83±0.05b	1.05±0.09a	0.91±0.61A	1.16±0.09A
		tobacco	-	-	-	-	-
30	8,11,14-20:3	spruce	0.01±0.01c	0.08±0.02b	0.19±0.01a	0.06±0.01B	0.15±0.01A•
		tobacco	-	-	-	-	-
32	21:0	spruce	-	-	-	-	-
		tobacco	0.05±0.03a	0.04±0.01a	0.03±0.01a	0.03±0.01A	0.04±0.02A

33	22:0	0.86±0.14a	1.20±0.51a	0.59±0.13a	0.68±0.06A	0.88±0.24A
		0.87±0.09a	0.83±0.14a	0.93±0.03a	0.87±0.01A	1.01±0.26A
36	23:0	-	-	-	-	-
		0.08±0.03a	0.06±0.02a	0.07±0.03a	0.05±0.02A	0.03±0.01A
37	24:0	0.76±0.12a	1.11±0.49a	0.66±0.09a	0.78±0.07A	1.39±0.79A
		0.93±0.08a	0.61±0.35a	0.99±0.09a	0.91±0.18A	0.97±0.22A
38	25:0	0.07±0.03a	0.16±0.07a	0.02±0.01b	0.08±0.01A	0.13±0.08A•
		0.01±0.01a	0.01±0.01a	0.01±0.01a	-	-
39	26:0	0.16±0.03a	0.24±0.08a	0.19±0.07a	0.15±0.05A	0.28±0.14A
		0.01±0.01a	0.02±0.01a	-	-	-
41	28:0	0.15±0.12a	0.47±0.40a	0.10±0.04a	0.22±0.02A	0.34±0.20A
		-	-	-	-	-
42	30:0	0.01±0.01	-	-	0.01±0.01A	0.03±0.01A
		-	-	-	-	-

The data are presented in tables and a graph as the means ± SEM. Statistical analysis was performed using one-way ANOVA followed by post hoc analysis using Tukey's honest significant difference (HSD) for unequal N tests. Different letters and symbols show significantly different values. Mean values were considered significantly different at $P < 0.05$. Analysis was performed separately for medium, medium+H_2O_2, and for stage of germination or growth.
